# Breast myopathy co-occurrence and its impact on carcass and meat quality attributes in broiler chickens

**DOI:** 10.1016/j.psj.2024.104625

**Published:** 2024-12-03

**Authors:** Míriam Muñoz-Lapeira, Maria Font-i-Furnols, Albert Brun, Anna Jofré, Marcos Botella, Cristina Zomeño

**Affiliations:** a IRTA-Food Safety and Functionality, Finca Camps i Armet, Monells, Spain; b IRTA-Food Quality and Technology, Finca Camps i Armet, Monells, Spain; c Vall Companys Group, Lleida, Spain; d Departamento de Producción y Sanidad Animal, Salud Pública Veterinaria y Ciencia y Tecnología de los Alimentos, Facultad de Veterinaria, Universidad Cardenal Herrera-CEU, CEU Universities, Alfara del Patriarca, Spain

**Keywords:** Myopathy combination, Chemical composition, Discriminant analysis, Poultry meat, Technological quality

## Abstract

Wooden breast (WB), spaghetti meat (SM) and white striping (WS) are significant research focuses due to their impact on meat quality. This study examines the relationship between different myopathies in chickens from a commercial slaughterhouse and compares physicochemical traits between breasts with single and multiple myopathies and control (CO-no apparent myopathy). A total of 240 breasts were evaluated for myopathy presence, severity and location, and physicochemical parameters (i.e. carcass weight and color; breast color, pH, electrical conductivity, drip, thaw and cooking loss, Warner-Brazler texture, moisture, protein, fat and collagen content). A 54.8 % of the affected breasts presented multiple myopathies, and within them, a correlation between WB and WS was found (Kendall's tau = 0.24; *P <* 0.01). Additionally, myopathies were more prevalent in the breast cranial region (*P <* 0.05). Most physicochemical parameters varied significantly across myopathy classes. Breasts presenting WB, alone or in combination, were the most altered, showing: heavier carcasses with higher breast yield and redder and yellower skin; breasts with higher L*, b* and Hue, higher moisture, fat and collagen and lower protein content, and with higher cooking loss and lower resistance to shear (*P <* 0.05). SM, WS and their combination often had intermediate values between CO and WB breasts, with a few exceptions in carcass color and breast fat content. The principal component discriminant analysis revealed a proximity of CO to WS, SM, and their combinations, and a clear separation to WB and WB-SM. Breast yield, pH, cooking and thaw loss, and protein and fat content were the most discriminative parameters between categories. The partial least squares discriminant analysis could not differentiate between single, multiple myopathies and CO (accuracy = 42.6 %), but showed 80.63 % balanced accuracy for WB-SM, 74.26 % for SM and 74.61 % for CO. These findings confirm most previously reported data on meat quality, and provide a thorough analysis that can help industries to improve breast myopathies postmortem classification and identification.

## Introduction

Poultry is one of the most popular meats globally, and its consumption is projected to continue increasing (OECD/FAO, 2024). In developed countries, it is gaining popularity due to nutritional and dietary properties. Consumers are expecting responsible production practices, both environmentally and in terms of animal welfare, while maintaining a high quality (Vukasovic and Stanton, 2017). In developing countries, it serves as a crucial protein source due the absence of religious restrictions and its cost-effective production (Pica-Ciamarra and Otte, 2010).

Due to the rising demand and the pursuit of increased profits, poultry has undergone extensive genetic selection to enhance growth rate and breast yield. However, this has led to a surge in white striping (WS), wooden breast (WB) and spaghetti meat (SM) myopathies. Despite the distinct physical appearances of these myopathies, their etiology appears to be related, with similar histological lesions (Baldi et al., 2018) and described alterations in metabolic pathways. These alterations involve hypoxia, oxidative stress and disrupted redox homeostasis, leading to inflammation, myodegeneration, fibrosis and lipidosis (Zhang et al., 2023; Pesti-Asbóth et al., 2023; Alnahhas et al., 2023). These muscle abnormalities significantly devalue the product on several dimensions. Firstly, myodegeneration, lipidosis and fibrosis impact their texture and visual appearance, decreasing their sensory qualities for the consumer (Petracci et al., 2019). Moreover, their nutritional qualities are also impacted, with lower protein content and higher moisture and fat, especially reported on WB and WS. Known technological impairments include increased final pH and exudation, and changes in color (Tasoniero et al., 2016; Baldi et al., 2018).

This situation is causing substantial economic losses (Barbut, 2019), making the understanding of myopathies a current focus for the industry. Some studies have documented breasts affected by multiple myopathies (Che et al., 2022a; 2022b; Wang et al., 2023), with an incidence as high as 85.1 % among affected breasts. However, to our knowledge, an exhaustive statistical evaluation of the association between these myopathies has not been conducted. Besides, breasts affected by myopathies share some changes in quality traits but differ in others. This hinders a correct evaluation and identification of affected breasts, impeding producers to detect this meat early in the slaughter chain and to find its best final destination. While reviews of published physicochemical analyses on chicken with myopathies have been conducted (Soglia et al., 2019a; Petracci et al., 2019; Barbut, 2019; Bošković et al., 2023), gaps, especially in SM alterations and in the combined effect of multiple myopathies, remain to be filled. Consequently, it is crucial to perform a complete characterization of affected breasts (with one or multiple myopathies) that allows to elucidate differences in their organoleptic, technological and nutritional traits, identifying possible interactions between the effects of different myopathies. A detailed knowledge of the effect that myopathies have on the chicken breast would also help to develop objective and standard methods to detect these abnormalities.

Therefore, the aims of this study were (1) to assess the relationship between the occurrence of WS, WB, and SM myopathies in chicken broilers obtained from a large commercial slaughterhouse, and (2) to compare carcass and meat physical and chemical characteristics between breasts with single and multiple myopathies and control breasts (no apparent myopathy), elucidating how myopathies interact and which parameters they alter.

## Materials and methods

### Sampling and sorting procedure

Carcasses: In a commercial slaughterhouse, 240 chicken carcasses were collected, 60 for each myopathy group (WS, WB, SM) and a control group (CO), without any evident myopathies, and transported to IRTA facilities with a refrigerated truck (4 °C) within 8 h after slaughter. This assignment was performed at carcass level and with minimum invasion, allowing to assign only one myopathy or the absence of an evident one. This process occurred over 12 sampling batches, covering diverse flocks with animals of different ages (39.6 ± 1.4 days), sexes (males and females) and slaughter weight (2.70 ± 0.41 kg), from different farms, and during different seasons (June to November 2022) to achieve maximum variability and representativeness of the slaughtered population. All chickens belonged to a commercial and fast-growing strain (Ross 308).

Once arrived, carcasses were thoroughly evaluated by two trained members of the research team to confirm the myopathy presence, to check co-occurrence of myopathies, and to score its severity and assess its location. The criteria defined by Kuttappan et al. (2012), Petracci et al. (2019), Wold et al. (2017) were used with some modifications.

Breasts: Deboned breasts presenting white stripes diffused across the breasts and narrower than 1 mm were scored moderate WS, while those wider than 1 mm were scored severe. Breasts with white stripes <1 mm appearing locally were not considered WS given the high prevalence and industrial acceptance of this abnormality. Moderate WB was assigned if hardness and pale patches with or without petechiae were observed locally, and it was scored severe if it affected 75 % of the breast. SM was scored moderate when fiber separation was observed locally or only detected through palpation (not visually evident), and severe when the lesion was extended and the fiber separation was observable at sight. These criteria were followed to assign a global class for each breast, making a total of 8 different categories. Table 1 summarizes this final assignment and compares it with the given category at slaughterhouse. In total, of the 240 breasts, 41 were CO, 31 WS, 7 WB, 52 SM, 22 WB-WS, 15 WB-SM, 42 SM-WS, and 30 WB-SM-WS.

**Table 1:** Summary of the samples selected according to myopathy class. On columns, the original classification made on the slaughterhouse and the breast region classification made at IRTA facilities. On rows, the final classification at global breast level made at IRTA facilities. Control (CO), white striping (WS), wooden breast (WB), spaghetti meat (SM) and their combinations were classified at global breast level.

	Classification at slaughterhouse				Breast region		
	CO (*n =* 60)	WS (*n =* 60)	WB (*n =* 60)	SM (*n =* 60)	Cranial (*n =* 240)	Central (*n =* 240)	Caudal (*n =* 240)
CO (*n =* 41)	34	6	1	0	3	49	64
WS (*n =* 31)	5	26	0	0	72	92	57
WB (*n =* 7)	0	0	7	0	0	8	12
SM (*n =* 52)	18	7	0	27	3	12	43
WB-WS (*n =* 22)	0	2	19	1	26	53	16
WB-SM (*n =* 15)	0	1	13	1	1	0	5
SM-WS (*n =* 42)	3	13	2	24	107	19	38
WB-SM-WS (*n =* 30)	0	5	18	7	28	7	5

**Fig. 1:**
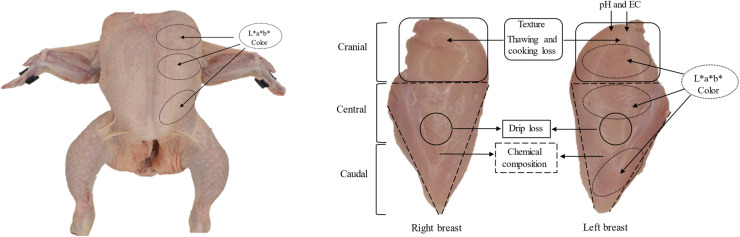
Graphical representation of the carcass and breast sampling sites. In brackets the three regions (cranial, central, and caudal) used to virtually divide the breasts.

As shown in Fig. 1, every breast was virtually divided into three regions: cranial, which corresponded to the first third of the upper part of the breast; central, which corresponded to the middle part; and caudal, which corresponded to the lower third part of the breast. Breasts were also characterized in these three anatomical regions individually with the presence of each myopathy, making the same 8 different categories used for the global breast classification. Table 1 summarizes the myopathy categories in each region.

### Physical determinations

Fig. 1 summarizes the carcass and meat measurements performed and their location. Skin carcass color was measured in the cranial, central, and caudal regions of the left breast, avoiding obvious defects such as hemorrhages or liquid. Measurements were taken in triplicate using a portable spectrophotometer Minolta CM-600d (Minolta Inc., Tokyo, Japan) with standardized conditions (illuminant D65, observation angle of 10° and an aperture of 8 mm) and determining: lightness (L*), redness (a*) yellowness (b*), and chroma and hue angle values.

Carcasses were then weighed and deboned. Once breasts were obtained, they were weighed, and *Pectoralis minor* muscle was removed so that all the meat quality determinations were made in the *Pectoralis major* muscle. Muscle color (L*, a*, b*, chroma and hue angle) was measured in the dorsal position of the left breast. Three regions (cranial, central, and caudal) were scanned in triplicate and avoiding obvious defects as described for the carcass measures. Muscle pH and electrical conductivity were determined in duplicate in the antero-dorsal position of the left breast (cranial region, Fig. 1). A portable pHmeter with a Xerolyt electrode (Crison, Barcelona, Spain) with automatic temperature compensation function was used for the pH determinations. The Pork Quality Meter (PQM-Kombi, Aichach, Germany) device was used for the electrical conductivity measurements.

Drip loss was measured immediately in two portions (from left and right breasts, respectively) of the central region following the method EZ drip loss (Rasmussen and Andersson, 1996). Two standardized 2.5 cm diameter round cylinders were obtained using a hand-held coring device and kept in a closed meat container at 4 °C for 24 h. The remaining meat of the central and caudal regions (from left and right breasts) was grinded, vacuum packed and stored at −20 °C until the chemical analysis. The whole cranial regions (from left and right breasts) were vacuum packed and stored at −20 °C until texture analysis was performed. Chemical and texture determinations were performed approximately 30 days later.

Thawing and cooking loss were measured in duplicate in the same breast regions where texture was analyzed (Fig. 1). Thawing loss was calculated after maintaining the samples at 4 °C for 24 h and removing water excess with soft touches of absorbent paper. Cooking loss was calculated after cooking the thawed portions in plastic bags in a water bath at 77 °C until reaching a core temperature of 75 °C, measured with a penetration prove, and cooling down for one hour. The water loss (drip, thaw and cook) was expressed as the weight difference regarding the initial sample weight in percentage.

The Warner Bratzler shear force analysis was conducted on six 1.25 cm diameter cylindrical cores, obtained using a hand-held coring device and cut parallel to the longitudinal orientation of muscle fibers. A TA.TX2 texture analyser (Aname, Stable Micro Systems, Godalming, United Kingdom) equipped with a 30-kg load cell and a shear V shaped blade set at a crosshead speed of 2 mm/s was used to measure: the maximum shear force, leading to the maximum force required to shear across the meat; the area under the recorded curve (total shear force), indicating the total energy required to shear across the meat; and the slope of the recorded curve between 20 % and 80 % of the maximum force (slope force).

### Chemical determinations

Moisture, protein, fat, and collagen content were analyzed in duplicate, and following official methods (AOAC, 2000). The moisture content was determined by drying the sample in an oven at 103°C until constant weight was achieved. The determination of the protein content was based on the Kjeldahl total nitrogen content using a nitrogen/protein analyzer (Digestion Unit K-435 and Scrubber K-415, BÜCHI, Flawil, Switzerland). The fat content was determined by solvent extraction with petroleum ether (Fat Extractor E-500, BUCHI, Flawil, Switzerland) with a pre-extraction acid hydrolysis treatment.

The total collagen content was determined according to hydroxyproline method as described by Petracci and Baeza (2011) with some modifications. Briefly, breast samples were hydrolyzed in sulphuric acid at 105 °C, filtered and diluted. After oxidation with chloramine-T and dilution, hydroxyproline content was calculated by determining the color absorbance at 558 nm wavelength. Then, a factor of 7.25 was used to convert hydroxyproline content to total collagen content.

### Statistical analysis

All analyses were performed using R software (v4.3.0; R Core Team, 2022).

Chi-squared tests were used to evaluate the independence between myopathies using two approaches. On the first, each myopathy was presented as a logical variable (if present: true; if absent: false). On the second, the myopathy was presented as a categorical variable (0: absence; 1: moderate; and 2: severe). For each single myopathy and myopathy combination, expected frequencies under the assumption of independence were calculated and compared with observed frequencies. Pearson residuals higher than 2 were considered to indicate a significant difference between expected and observed values. A plot to visualize the results was generated with the mosaic function in the vcd package (v1.4-12; Zeileis et al., 2007).

Pairwise Kendall's rank correlation between myopathies (WB and WS, WS and SM, and SM and WB) were calculated using myopathy severity (categorical variable approach).

Another chi-square test was used to investigate if myopathy presence was random across the breast. To do that, the instances of myopathy presence by breast region (cranial, central, or caudal) were calculated under the assumption of independence and compared with observed frequencies. Pearson residuals higher than 2 were considered to indicate a significant difference between expected and observed values. A mosaic plot to visualize the results was also used (v1.4-12; Zeileis et al., 2007).

A general linear model incorporating myopathy presence and sampling day as fixed effects was applied for each physical and chemical attribute. Myopathy presence included both breasts presenting one and multiple myopathies. Logarithmic correction was applied for drip loss and texture parameters to transform their distribution from power law to normal, while a range correction was implemented for color variables to normalize extreme values, enhancing interpretability and comparability. Least squared means were compared for myopathy presence using a Tukey test where a *P* value equal or higher than 0.05 was considered to indicate a significant difference (v2.30-0; Lenth, 2016).

Additionally, to quantify and visualize the physical and chemical alterations, a Discriminant Analysis of Principal Components (DAPC) was conducted on normalized and centered physicochemical variables. The variables selected for analysis included all the significantly different variables from the general linear model analysis, along with all color parameters from the breast cranial and central regions (v2.1.20; Jombart and Ahmed, 2011). Principal components representing 90 % of the variance were used. To test the discriminant power of the variables, a Partial Least Squares Discriminant Analysis was carried out, using complete cross-validation (10-fold, 20 repeats) to assess the accuracy (v6.0-94; Kuhn, 2008).

## Results and discussion

### Breast myopathy presence and association

Breasts presenting multiple myopathies represented 54.8 % of the affected breasts, with 15.1 % of them showing three myopathies. Che et al. (2022a) evaluated chicken breasts from two processing plants and found an even higher proportion, with 85.1 % of the fillets presenting multiple myopathies and 32.8 % presenting all three. In another study, Che et al. (2022b) found similar results, with 68.2 % of the samples affected by more than one myopathy.

**Fig. 2:**
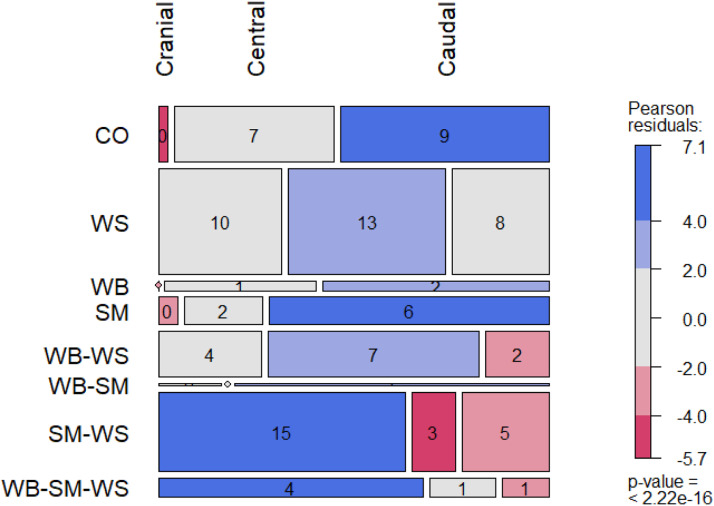
Mosaic plot representing a contingency table between anatomical breast regions (columns) and myopathy classes (rows). The height of each tile expresses the proportion of myopathy observations and the width the proportion of regions. Numbers inside each tile are the observed proportion, in percentage, of each combination. Pearson residuals measure the difference between the observed and expected frequencies: red tiles indicate lesser presence than expected and blue tiles greater. CO: control; WB: wooden breast; SM: spaghetti meat; WS: white striping; WB-WS: co-occurring WB and WS; WB-SM: co-occurring WB and WM; SM-WS: co-occurring SM and WS; WB-SM-WS: co-occurring WB, SM and WS.

Fig. 2 represents a mosaic plot showing the relationship between myopathies and breast anatomical regions. The height of each tile expresses the proportion of myopathy observations, and the width the proportion of regions. Colored tiles indicate deviations from independence (Pearson residuals > 2 or <-2). Positive residuals, in blue, indicate higher observed counts than expected, while negative residuals, in red, represent lower counts. Generally, the cranial region (first column of tiles) was the most affected by myopathies. In this region, CO breasts (first row of tiles), i.e. meat without any apparent part affected, were observed less frequently than expected (narrow and red-colored tile), while SM-WS and the triple combination were found more frequently than expected (wider and blue-colored tiles). Conversely, the caudal region was the least affected, with a wider and blue-colored tile for CO breasts, indicating a greater proportion of unaffected meat, while SM-WS and the triple combination were less observed (narrow and red-colored tiles). In addition, the single SM and WB conditions were found more frequently than expected (wider and blue-colored tile) in this region, although at lower proportions with respect to the global myopathy assignments (tiles with lower height). WB-SM were also observed in low proportion (low height), and almost entirely in the caudal region (widest third column). Lastly, WS and WB-WS myopathies were found more frequently than expected in the central region (wider and blue-colored tiles).

This distribution is in accordance with the observations of other authors, that have pointed out that myopathies occur, or are more severe, on the upper layers of the cranial region (Soglia et al., 2017; Tasoniero et al., 2017; Wold et al., 2017; 2019). This is also true when observing the occurrence of multiple myopathies, as done in this study; combinations are more likely to manifest in the cranial and central regions.

**Fig. 3:**
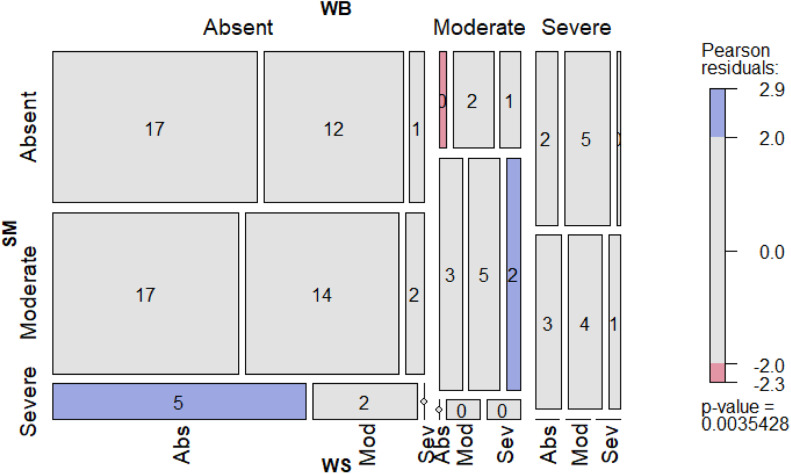
Mosaic plot representing a three-way contingency table between the absence or presence in moderate or severe manifestation of wooden breast (WB, upper horizontal), spaghetti meat (SM, vertical) and white striping (WS, lower horizontal) classified at global breast level. The size of each tile represents the observed proportion of each possible myopathy combination, and the number, its percentage. Pearson residuals measure the difference between the observed and expected frequencies: red tiles indicate lesser presence than expected and blue tiles greater.

Fig. 3 represents a mosaic plot associating myopathies and severity degree. Each tile expresses the absence or presence in moderate or severe manifestation of each myopathy (WB, SM and WS), scored in the global breast. Breasts presenting moderate WB in absence of WS and SM occurred less frequently than expected (frequency observed: 0.42 % vs. frequency expected: 3.02 %), while myopathies presenting only severe SM, in the absence on WS and WB, occurred more frequently than expected (5.00 % vs. 2.97 %). Additionally, the triple combination of moderate WB, moderate SM, and severe WS occurred more frequently than expected (2.1 % vs 1.8 %).

Evaluating their independence, the chi-squared test between WB and WS presence revealed a significant association between the myopathies (*P <* 0.001). However, no relationship between SM and WB or between SM and WS (*P >* 0.05) was found. Kendall's correlation rank confirmed the relationship between WB and WS, with a value of 0.24 (*P <* 0.01). It is important to highlight that these associations could be partially explained by the difference in sex occurrence, since WS and WB have been more reported in males (Kuttappan et al., 2013; Lorenzi et al., 2014; Trocino et al., 2015), while SM in females (Soglia et al., 2019a), and samples of this study were not balanced by sex within myopathy. Che et al. (2022a) reported a high amount of myopathy combinations in breast fillets from 37 flocks and two processing plants. Using the data provided by these authors, a Kendall's correlation between WS and WB of 0.34 was obtained (calculations made by the authors of the present study). It should be noted that, in the study of Che et al. (2022a), mild WS (white stripes < 1mm and appearing locally) was considered as an alteration, and its presence in 93.8 % of the analyzed breasts likely increased the correlation value. Significant associations between SM and WS and between WB and SM were also found, although Kendall's correlation values were low (0.12 and 0.17, respectively, calculated by the authors of the present study).

The results of the present study support the theory that WB and WS are manifestations of the same underlying disease (Sihvo et al., 2017), consisting of a replacement of the muscle structure with adipose tissue or thickening of the connective tissue layers (Kuttappan et al., 2016; Sihvo et al., 2017). On the other hand, SM was found to be independent or to have low correlation, since this abnormality is more related to the thinning of the connective tissue, that becomes insufficient to maintain muscle fibers adhered (Baldi et al., 2018). Moreover, it has been recently proposed that postmortem processing steps, such as defeathering, deskinning, and deboning may be involved in the appearance or exacerbation of SM (Bailey, 2023; Baldi et al., 2021; Che et al., 2022a).

### Carcass and meat physical quality comparison

Supplementary tables summarize the comparisons between all single myopathies and myopathy combinations for carcass and breast physical parameters (Supplementary Tables 1 and 2, respectively) and for breast chemical composition (Supplementary Table 3). WB-WS were not different from WB for any parameter, unlike WS, which were different from WB-WS in some instances. Similarly, WB-SM-WS were not different from WB-SM for any parameter, except for fat content, but they were different from SM-WS in the rest of the parameters evaluated.

As a result, and in order to have a better-balanced dataset, WB (*n =* 7) and WB-WS (*n =* 22) were merged and considered as WB. WB-SM (*n =* 15) and WB-SM-WS (*n =* 30) were also merged and considered as WB-SM. The following results and discussion explore the differences between these simplified myopathy class; comparing WB (*n =* 29), WS (*n =* 31), SM (*n =* 52), SM-WS (*n =* 52), WB-SM (*n =* 45) and CO (*n =* 41).

Table 2, Table 3 present carcass and breast physical parameters, respectively, according to myopathy class. Significant differences were observed among myopathy classes for nearly all attributes. Generally, the presence of WB, either alone or in combination with SM (WB-SM), led to alterations compared to CO. In addition, SM-WS condition was usually similar to single SM and WS, although each class (SM-WS, SM and WS) showed distinctive significant differences when compared to other myopathy classes.

**Table 2:** Carcass physical parameters (least square mean) according to myopathy class. Control (CO), white striping (WS), wooden breast (WB), spaghetti meat (SM), and combinations WB-SM and SM-WS were classified at global breast level.

	CO	WS	WB	SM	WB-SM	SM-WS	RMSE	*P* value
*Carcasses (n)*	*41*	*52*	*42*	*29*	*45*	*31*		
Weight (g)	2042^ab^	2193^bc^	2431^d^	2008^a^	2346^cd^	2100^ab^	217.85	<0.001
Breast yield (g/100 g carcass)	32.51^a^	33.59^ab^	35.37^c^	33.43^a^	34.89^bc^	33.65^ab^	1.81	<0.001
Cranial section color								
L*	67.64	67.83	68.88	68.24	68.99	68.16	2.52	0.174
a*	0.09^a^	0.47^ab^	1.26^bc^	0.56^ab^	1.54^c^	0.92^bc^	1.13	<0.001
b*	8.45^a^	8.28^a^	9.84^b^	9.63^ab^	10.78^b^	10.26^b^	1.91	<0.001
Hue	90.55^b^	87.29^ab^	84.02^a^	87.25^ab^	83.91^a^	86.79^ab^	5.98	<0.001
Chroma	8.46^ab^	8.29^a^	9.73abc	9.75^bc^	11.02^c^	10.46^c^	1.96	<0.001
Central section color								
L*	65.77	65.74	66.55	65.98	65.78	65.53	1.86	0.381
a*	-1.01^a^	-0.88^ab^	-0.16^c^	-0.80abc	-0.29^bc^	-0.95^a^	0.91	<0.001
b*	7.10	6.94	7.78	7.71	7.99	7.50	1.87	0.192
Hue	99.35^b^	97.99^ab^	92.91^a^	96.98^ab^	94.07^ab^	98.10^ab^	7.76	<0.01
Chroma	7.33	7.23	7.88	7.78	8.11	7.61	1.71	0.2984
Caudal section color								
L*	65.60	65.50	66.38	65.99	66.06	65.45	1.86	0.282
a*	-1.56	-1.50	-1.10	-1.51	-1.33	-1.39	0.86	0.306
b*	7.58	7.10	8.05	7.69	7.93	8.04	2.21	0.544
Hue	102.81	101.53	98.20	101.38	101.30	101.13	7.57	0.356
Chroma	7.84	7.47	8.28	7.89	8.22	8.27	2.03	0.576

RMSE = root mean square error

a,b,c different superscripts within row indicate significant differences between myopathy classes (*P* ≤ 0.05; Tukey test).

**Table 3:** Breast physical parameters (least square mean) according to myopathy class. Control (CO), white striping (WS), wooden breast (WB), spaghetti meat (SM), and combinations WB-SM and SM-WS were classified at global breast level.

	CO	WS	WB	SM	WB-SM	SM-WS	RMSE	*P* value
*Breasts (n)*	*41*	*52*	*42*	*29*	*45*	*31*		
Cranial region color								
L*	55.73^a^	56.11^ab^	57.83^b^	56.65^ab^	56.98^ab^	56.98^ab^	2.31	<0.01
a*	-0.54	-0.14	0.15	-0.37	-0.17	-0.54	0.98	0.060
b*	9.49^a^	10.52^ab^	13.27^d^	10.86^bc^	11.84^c^	11.32^bc^	1.51	<0.001
Hue	93.81^b^	91.39^ab^	88.27^a^	92.08^ab^	91.33^ab^	92.97^b^	5.40	<0.05
Chroma	9.54^a^	10.54^b^	12.35^c^	10.84^b^	11.92^c^	11.36^bc^	1.35	<0.001
Central region color								
L*	54.96	54.97	56.60	55.40	55.72	55.44	2.28	0.061
a*	-1.49^a^	-1.19^a^	-1.01^a^	-1.45^a^	-1.10^a^	-1.43^a^	0.70	<0.05
b*	6.99^a^	7.72^ab^	8.32^b^	7.55^ab^	7.99^b^	7.74^ab^	1.46	<0.01
Hue	102.27^a^	98.85^a^	98.13^a^	101.69^a^	98.83^a^	101.32^a^	5.99	<0.05
Chroma	7.21^a^	7.91^ab^	8.46^b^	7.77^ab^	8.20^b^	8.01^ab^	1.46	<0.05
Caudal region color								
L*	55.67	54.71	56.29	56.05	54.86	55.35	2.45	0.061
a*	-1.34	-1.52	-1.67	-1.46	-1.42	-1.45	0.62	0.426
b*	7.37^a^	7.04^a^	8.02^a^	7.74^a^	7.57^a^	7.94^a^	1.29	<0.05
Hue	101.14	103.07	102.72	101.43	101.73	101.13	5.60	0.647
Chroma	7.54 a	7.28 a	8.26 a	7.96 a	7.84 a	8.19 a	1.31	<0.05
pH	5.78^a^	5.87^a^	6.02^b^	5.78^a^	6.02^b^	5.87^a^	0.148	<0.001
EC	8.12abc	8.05abc	9.36^c^	7.73^ab^	8.88^bc^	7.49^a^	1.947	<0.001
Drip loss ( %)	1.38	1.25	1.84	1.56	1.46	1.38	1.75	0.121
Thawing loss ( %)	11.54^bc^	10.29abc	9.74^ab^	11.82^c^	8.76^a^	11.50^bc^	2.840	<0.001
Cooking loss ( %)	25.40^a^	26.10^a^	31.75^c^	26.72^a^	29.43^b^	26.91^a^	2.699	<0.001
Warner-Brazler texture								
Maximum force (N)	17.24^c^	15.83abc	14.36^ab^	16.35^bc^	13.42^a^	15.92^bc^	1.28	<0.001
Total force (N × mm)	60.00^b^	54.63^bc^	49.19^ab^	56.92^bc^^bc^	44.83^a^	54.50^ab^	1.32	<0.001
Slope force (N/mm)	3.01^b^	2.81^ab^	2.75^ab^	2.92^ab^	2.54^a^	2.84^ab^	1.26	<0.05

RMSE = root mean square error; EC = electrical conductivity

a,b,c different superscripts within row indicate significant differences between myopathy classes (*P* ≤ 0.05; Tukey test).

Carcasses were heavier in WB and WB-SM class (2431 and 2346 g, respectively) than in SM, SM-WS, and CO (2008-2100 g), while WS showed intermediate weights (2193 g) and significantly different from WB and SM (Table 2). For breast yield, WB had a higher proportion (35.3 %) compared to WS, SM, SM-WS and CO groups (32.5-33.6 %), while WB-SM showed intermediate yield values (34.8 %) and similar to WB, WS, and SM-WS classes. Sex-related myopathy distribution, with WB being more prevalent in males (Trocino et al., 2015; Bordignon et al., 2022), which are heavier, and SM in females (Soglia et al., 2019a), which are lighter, may explain the observed weights.

Color indexes varied across myopathy classes, particularly in the cranial region of the carcass and breast (Table 2, Table 3 respectively), coinciding with the portion where myopathies were more likely to appear. Regarding the carcass, the red index (a*) was higher in both the cranial and the central regions of WB and WB-SM compared to CO (Table 2). SM-WS carcasses were redder than CO only in the cranial region. The increased redness might be due to the increased haemoglobin extravasation described in myopathies (Alnahhas et al., 2023). Also in the cranial region, higher b* values were shown by WB, WB-SM, and SM-WS compared with WS and CO, possibly due to a higher accumulation of feed pigments, which are liposoluble compounds, in the subcutaneous fat, as described by Campo et al. (2020).

Hue values reflected the observed differences in redness and yellowness. In the cranial region of the carcass, CO samples showed typical yellow values (90.55), while WB and WB-SM were redder (84.02 and 83.91, respectively) (Munsell, 1905; Table 2). In the central region, differences between CO (99.34, greener) and WB (92.91, yellower) were seen. Chroma values only differed in the cranial region of the carcass, where WB-SM (11.02) and SM-WS (10.46) presented stronger colors than CO (8.46) and WS (8.29).

On the skinless breast, WB exhibited higher L* and b* indexes compared to CO in the cranial region (Table 3). Lighter and yellower meat in WB samples are in accordance with previous works (Campo et al., 2020; Wold et al., 2017; Wang et al., 2023), although in contrast with others (Zambonelli et al., 2016). Higher b* values were also shown by WB-SM, SM-WS and SM compared with CO. In this case, no differences were found between WS and CO, differing from other studies (Alnahhas et al., 2016; Campo et al., 2020). Higher b* values were also observed in the central region for WB-affected breasts (WB and WB-SM).

All myopathic breasts showed stronger colors (higher chroma) than CO ones in the cranial region, with WB, WB-SM and SM-WS showing higher values than SM and WS (Table 3). Higher chroma values were also observed in the central region for WB and WB-SM compared to CO. Although no significant differences in the redness index were found, hue values showed that WB were redder than SM and CO on the cranial breast (88.27 vs 92.97 and 93.81, respectively). In line with our results, Wang et al. (2023) found decreased chroma values on normal breasts compared with breasts with different combinations of myopathies, all including WB, and Campo et al. (2020) found increased chroma on breasts with myopathies (WB, WS and SM), without considering combinations.

Higher pH values were found in WB-affected breasts (6.02 in WB and WB-SM) compared to the other types (5.78-5.87) (Table 3). Single WS, SM or their combinations (SM-WS), however, did not show higher pH values than normal breasts. A reduced glycolytic potential in affected breasts has been suggested to explain the higher muscle pH (Kuttappan et al., 2017). Nevertheless, some studies did not find any relationship between myopathy presence and breast pH (Petracci et al. 2019; Bošković-Cabrol et al., 2023).

Electric conductivity, which relates to the presence of free water, varied across myopathy classes, with WB-affected breasts exhibiting higher values (8.88 in WB-SM and 9.36 in WB) than SM and SM-WS (7.73 and 7.49, respectively). Tasoniero et al. (2017) investigated molecular water movement in WB, and found increased extramyofibrillar water in WB, which can explain the increased electric conductivity in these samples.

No differences were observed in drip loss measurements, possibly due to the reduced water loses obtained (1.38-1.84 %) which may have limited the ability of this gravimetric method to accurately estimate them. Other studies showed contrasting results. For instance, Mudalal et al. (2014) found decreased drip loss in WS breasts while Alnahhas et al. (2016) recorded increased drip loss. Wang et al. (2023) calculated the drip loss storing the whole breast in plastic bags and observed an increase for fillets presenting triple myopathies and WB-WS compared to normal. This methodology could be more adequate for chicken breast samples since, although drip loss values were still low, it used a more representative sample.

When looking at water losses measured in thawed samples, WB-affected breasts showed the lowest values (8.76 % in WB-SM and 9.74 % in WB) and SM the highest (11.82 %), with SM-WS and CO in between but not significantly different from SM (Table 3). Differing results in thawing loss have been reported, from no effect on WS or WB to a decreased loss for WS (Petracci et al., 2019). At the time of cooking, process where the greatest water loss took place, WB breasts showed the highest values (31.75 %), followed by WB-SM (29.43 %), and then by the other breast types (25.40 %-26.91 %) with similar values among them (Table 3). These findings correlate well with those observed on electrical conductivity and with the impaired water holding capacity under heat treatment conditions described for myopathic meat (Petracci et al., 2019). The meta-study of Bordignon et al. (2022) reported a major impact of WB on cooking loss, with increased values in WB and WB combinations compared to control breasts.

Texture measurements reported in Table 3 revealed that WB-SM breasts required the least force to shear their fibers (maximum force = 13.42 N, total force = 44.83 N×mm, slope force = 2.54 N/mm), while CO breasts required the most (maximum force = 17.24 N, total force = 60.00 N×mm, slope force = 3.01 N/mm). A decreased maximum force was also found in WB compared to CO breasts, with no differences between the other classes. These results are inconsistent with previous studies reporting tougher meat in WB, WS, or WB-WS, on either raw, thawed, or cooked cuts (Trocino et al., 2015; Chatterjee et al., 2016; Tasoniero et al., 2016; Zambonelli et al., 2016; Bordignon et al., 2022). However, Bryon et al. (2020) found decreased Warner–Bratzler shear force for moderate and severe WB compared to normal meat after cooking the samples in an oven wrapped in aluminum foil. Sanchez-Brambila et al. (2018) also found softer meat on WB compared to control after cooking the vacuum-packed samples in a steam oven and using a Warner–Bratzler device. In addition, Petracci et al. (2013) showed decreased shear force in WS breasts measured with allo-Kramer, and Campo et al. (2020) found a tendency of WS being less resistant to Warner–Bratzler shear force, both in vacuum-packaged meat cooked in a water bath.

The lowest hardness found in WB-affected breasts compared to normal ones can be explained by different mechanisms and reasons. First, samples were submitted to a freezing storage with subsequent thawing, and it is known that this procedure alters the fiber muscle structure predominantly in myopathic meat (Soglia et al., 2019b). Second, the cooking process applied in the water bath at 75 °C had surely an impact on the muscle components, especially on the connective tissue which is predominantly altered by the presence of the WB condition (Petracci et al., 2019). As stated by Soglia et al. (2017), the thermally labile nature of the connective tissue cross-links explains the similar compression and shear force values they measured on WB and normal cooked meat. Campo et al. (2020) also pointed out the higher solubility of newly formed connective tissue when heat is applied. In fact, according to these authors, texture differences in the myopathies are better detected in raw than in cooked meat (Soglia et al. 2017; Campo et al., 2020). In addition, the different distribution of myopathies between deeper and superficial layers should be taken into account when assessing texture since the inclusion of all muscle structure changes in the sampling may be hindered. In this line, Sanchez-Brambila et al. (2018) found dorsal and ventral layers of WB to have different texture characteristics when evaluated using a sensory panel.

### Chemical quality comparison

Table 4 presents the results of breast chemical composition (moisture, protein, fat, and total collagen content). Breasts affected with WB (WB and WB-SM) were observed to have the most pronounced variations, with higher moisture, fat and collagen, and lower protein content than CO. SM and SM-WS classes showed in general similar characteristics to CO. The highest moisture (78.63 g/100 g) and the lowest protein content (18.86 g/100 g) was seen on WB, followed by WB-SM (77.77 and 19.67 g/100 g, respectively). WB-SM had the highest fat values (1.74 g/100 g), and WS and WB (1.60 and 1.69 g/100 g) also showed increased fat content compared with CO (1.26 g/100g). SM-WS (1.45 g/100 g), although not different than CO, showed significantly more fat than SM (1.15 g/100g). Higher collagen content was observed in WB-affected breasts (5.48 and 5.03 mg/g) compared to CO and SM (4.42 and 4.11 mg/g). WS (4.49 mg/g) had a lower collagen content than WB, but comparable to the other classes.

**Table 4:** Breast chemical composition (least square mean) according to myopathy class. Control (CO), white striping (WS), wooden breast (WB), spaghetti meat (SM), and combinations WB-SM and SM-WS were classified at global breast level.

	CO	WS	WB	SM	WB-SM	SM-WS	RMSE	*P* value
*Breasts (n)*	*41*	*52*	*42*	*29*	*45*	*31*		
Moisture (g/100g)	76.34^a^	76.50^a^	78.63^c^	76.65^a^	77.77^b^	76.89^a^	0.907	<0.001
Protein (g/100g)	21.64^d^	21.07^cd^	18.86^a^	21.33^cd^	19.67^b^	21.00^c^	0.954	<0.001
Fat (g/100g)	1.26^ab^	1.60^cd^	1.69^cd^	1.15^a^	1.74^d^	1.45^bc^	0.391	<0.001
Collagen (mg/g)	4.42^a^	4.49^ab^	5.48^c^	4.11^a^	5.03^bc^	4.49^ab^	0.013	<0.001

RMSE = root mean square error

a,b,c different superscripts within row indicate significant differences between myopathy classes (*P* ≤ 0.05; Tukey test).

Similar global alterations were noted in other studies, although inconsistencies are found particularly in moisture and collagen content (Petracci et al., 2019), which has been found to be different for WS and SM compared to CO (Mudalal et al., 2014; Petracci et al., 2014; Baldi et al., 2018). Differences in methodology among studies can be responsible for different results. In addition, the heterogenous distribution of myopathies both across regions and in muscle depth can add variability to the results.

### Physical and chemical quality relationships between myopathies

**Fig. 4:**
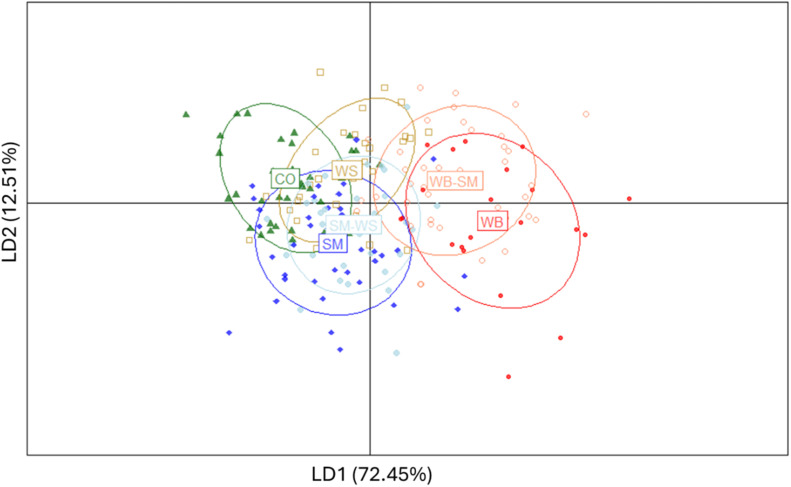
Discriminant analysis of principal components (DAPC) on scaled and centered physical and chemical variables. Myopathy class label is placed on the centroid of class observations. Ellipses represent 95 % confidence intervals for clusters. Control (CO), white striping (WS), wooden breast (WB), spaghetti meat (SM), and combinations WB-SM and SM-WS were classified at global breast level.

A Discriminant Analysis of Principal Components (DAPC) was conducted to synthesize the differences between myopathies and evaluate the distinctive power of the tested variables. The first two components of the discriminant linear functions (LD) captured 85.78 % of the variance. Fig. 4 displays the samples arranged in a two-dimensional space, with the x-axis (LD1) separating CO from WB and WB-SM breasts, and the y-axis (LD2) tending to separate CO from SM and SM-WS breasts. On a global view, the WS point cloud overlapped with all the other ellipses, indicating a progression of alterations in which this would be the intermediate step. SM overlapped with CO, indicating that physicochemical differences between SM and normal breasts are not evident, in line with the results of physicochemical traits. WB were the most different, and thus, the less overlapped cluster. Notably, CO samples showed less variation, indicating more stable parameters compared to the myopathic breasts.

Table 5 summarizes variable contributions, determined by assessing loadings on principal components. Breast yield and pH were the most contributing variables on LD1, followed by cooking loss, protein content, and carcass weight. On the second function, thawing loss, fat content, breast cranial yellow index (b*) and Hue, and carcass weight were the most informative. Texture parameters, electric conductivity and other color measurements contributed the least to discriminating between classes. Bordignon et al. (2022) also found WB to be the most altered myopathy when looking at pH, color, cooking losses and shear force, and Wang et al. (2023) considers WB-SM to be the most detrimental myopathy among different combinations and severities of WS, WB and SM. However, no study has evaluated the alterations jointly.

**Table 5:** Contribution of physical and chemical characteristics to each discriminant function (LD).

Variable	LD1 (72.45 %)	LD2 (12.51 %)
Breast yield (g/100 g carcass)	0.136	0.002
pH	0.134	0.001
Cooking loss ( %)	0.105	0.000
Protein (g/100g)	0.101	0.002
Carcass weight (g)	0.089	0.092
Fat (g/100g)	0.089	0.210
Hue cranial breast region	0.064	0.129
b* cranial breast region	0.062	0.128
L* cranial breast region	0.060	0.011
Moisture (g/100g)	0.055	0.021
Hue central breast region	0.023	0.014
b* central breast region	0.022	0.016
Collagen (mg/g)	0.019	0.015
Thawing loss (%)	0.016	0.296
Maximum force (kg)	0.008	0.004
Total force (kg × mm)	0.005	0.003
L* central breast region	0.004	0.018
Chroma central breast region	0.003	0.025
Slope force (kg/mm)	0.003	0.003
Electrical conductivity	0.000	0.006
Chroma cranial breast region	0.000	0.006

Overall, breast yield, pH, and protein and fat content are key factors differentiating myopathies from control breasts, and cooking and thawing losses must be considered for their technological implications. These results can be useful to characterize myopathy attributes, but fall short of effectively discriminating between them. In conclusion, although visual and tactile characteristics of myopathies are consistent, the implications on their quality are a progressive and heterogeneous matter.

In this line, when conducting Partial Least Squares (PLS) analysis with all the 21 variables (Table 6), only a 42.6 % of accuracy was obtained with cross-validation. However, balanced accuracies on each individual class revealed acceptable performances for WB-SM, CO and SM (80.63 %, 74.61 % and 74.26 %, respectively). When observing the confusion matrix for predicted versus reference values, the majority of missed categories involved WB being predicted as WB-SM and WS being predicted as CO.

**Table 6:** Confusion matrix and statistics of Partial Least Squares discriminant model prediction using all the significant physical and chemical parameters in addition to breast cranial and central color parameters. Control (CO), white striping (WS), wooden breast (WB), spaghetti meat (SM), and combinations WB-SM and SM-WS were classified at global breast level.

		Reference					
Predicted		CO	WS	WB	SM	WB-SM	SM-WS
	CO	25	13	0	8	1	6
	WS	3	4	0	0	1	3
	WB	0	0	9	0	4	2
	SM	8	6	0	27	3	9
	WB-SM	0	5	11	1	29	5
	SM-WS	2	1	2	6	1	12
*Metrics*							
Sensitivity (%)		65.79	13.79	40.09	64.29	74.36	32.43
Specificity (%)		83.43	96.07	96.75	84.24	86.90	92.94
Balanced accuracy (%)		74.61	54.93	68.83	74.26	80.63	62.69

Overall, these findings demonstrate that the presence of WB is dominant over SM in altering the breast characteristics, as pointed in Bordignon et al. (2022), and that WS is the closest to CO breasts, being the least altering myopathy.

## Conclusions

A remarkable myopathy co-occurrence was reported in chicken breasts obtained in a large commercial slaughterhouse. The simultaneous myopathy presence was found to be dependent on the breast region, the cranial being the most affected one. In addition, a tendency of increased severity degree corresponding to multiple myopathy presence was observed. Alterations of diverse physicochemical carcass and meat characteristics were associated with different myopathies, with WB being the most detrimental, and often aggravated when SM was also present (WB-SM). This indicates that efforts to identify and palliate WB are an important focus. On the other hand, SM and WS were seen to have quality traits close to CO, and to present comparable characteristics when combined (SM-WS). The correlations found support a shared pathogenic mechanism between WS and WB, suggesting that joint characterization of these myopathies could be more appropriate. Conversely, the absence of a clear relationship between SM and the other myopathies and the lack of separation from control breasts in the discriminant analysis confirm additional causative pre and postmortem factors in contrast to WB or WS. This distinctive property of SM warrants further investigation for a more complete understanding of its etiology and accurate characterization. The thorough evaluation carried out in this work can potentially help poultry industries in setting up technologies to sort meat affected with myopathies.

## Disclosures

The authors declare that they have no known competing financial interests or personal relationships that could have appeared to influence the work reported in this paper.

## Appendix Supplementary materials

Image, application 1
