# Supplementary material for: Breast myopathy co-occurrence and its impact on carcass and meat quality attributes in broiler chickens
Source: Poult Sci. 2024 Dec 3;104(1):104625. doi: 10.1016/j.psj.2024.104625 (PMC11667685; doi:10.1016/j.psj.2024.104625)
Supplement: Supplementary file 1 [file mmc1.docx]

SUPPLEMENTARY MATERIAL

Supplementary Table 1. Carcass physical parameters (least square mean) according to myopathy class. Control (CO), white striping (WS), wooden breast (WB), spaghetti meat (SM), and combinations WB-WS, WB-SM, SM-WS and WB-SM-WS were classified at global breast level.

|  | CO | WS | WB | SM | WB-WS | WB-SM | SM-WS | WB-SM-WS | RMSE | *P* value |  |  |  |  |  |
| --- | --- | --- | --- | --- | --- | --- | --- | --- | --- | --- | --- | --- | --- | --- | --- |
| *Carcasses (n)* | *41* | *31* | *7* | *52* | *22* | *15* | *42* | *30* |  |  |  |  |  |  |  |
| Weight (g) | 2042^ab^ | 2193^bc^ | 2442^cd^ | 2009^a^ | 2427^d^ | 2358^cd^ | 2100^ab^ | 2340^cd^ | 218.79 | <0.001 |  |  |  |  |  |
| Breast yield (g/100 g carcass) | 32.52^a^ | 33.59^abc^ | 36.17^d^ | 33.45^ab^ | 35.10^cd^ | 35.27^cd^ | 33.65^abc^ | 34.68^bcd^ | 1.81 | <0.001 |  |  |  |  |  |
| Cranial section color | | | | | | | | | | | |  |  |  |  |
| L* | 67.64 | 67.83 | 68.88 | 68.24 | 68.92 | 69.31 | 68.16 | 68.83 | 2.52 | 0.34 |  |  |  |  |  |
| a* | 0.09^a^ | 0.47^ab^ | 1.22^bc^ | 0.56^ab^ | 1.27^bc^ | 1.25^abc^ | 0.92^bc^ | 1.68^c^ | 1.13 | <0.001 |  |  |  |  |  |
| b* | 8.45^a^ | 8.28^a^ | 9.62^b^ | 9.62^abc^ | 9.92^abc^ | 10.41^bc^ | 10.27^c^ | 10.97^c^ | 1.91 | <0.001 |  |  |  |  |  |
| Hue | 90.55^b^ | 87.29^ab^ | 87.07^ab^ | 87.32^ab^ | 82.98^a^ | 84.26^a^ | 86.78^ab^ | 83.72^a^ | 5.98 | <0.001 |  |  |  |  |  |
| Chroma | 8.46^ab^ | 8.29^a^ | 8.79^abc^ | 9.73^abc^ | 9.99^bc^ | 10.52^c^ | 10.47^c^ | 11.28^c^ | 1.96 | <0.001 |  |  |  |  |  |
| Central section color | | | | | | | | | | | |  |  |  |  |
| L* | 65.77 | 65.74 | 66.31 | 65.98 | 66.23 | 65.78 | 65.53 | 65.77 | 1.86 | 0.381 |  |  |  |  |  |
| a* | -1.01^a^ | -0.88^ab^ | 0.02^ab^ | -0.80^ab^ | -0.22^b^ | -0.23^ab^ | -0.95^ab^ | -0.32^ab^ | 0.91 | <0.001 |  |  |  |  |  |
| b* | 7.10 | 6.94 | 8.36 | 7.72 | 7.58 | 7.59 | 7.50 | 8.10 | 1.87 | 0.192 |  |  |  |  |  |
| Hue | 99.35^b^ | 97.99^ab^ | 91.96^a^ | 96.96^a^ | 93.23^a^ | 93.73^a^ | 98.11^a^ | 94.24^a^ | 7.80 | <0.05 |  |  |  |  |  |
| Chroma | 7.33 | 7.23 | 8.44 | 7.79 | 7.69 | 7.70 | 7.61 | 8.31 | 2.28 | 0.33 |  |  |  |  |  |
| Caudal section color | | | | | | | | | | | |  |  |  | 8.44 |
| L* | 65.60 | 65.50 | 66.14 | 65.98 | 66.46 | 66.17 | 65.45 | 66.00 | 1.86 | 0.49 |  |  |  |  |  |
| a* | -1.56 | -1.50 | -1.10 | -1.51 | -1.13 | -1.31 | -1.39 | -1.33 | 0.86 | 0.53 |  |  |  |  |  |
| b* | 7.58 | 7.10 | 8.19 | 7.69 | 8.00 | 8.27 | 8.04 | 7.77 | 2.21 | 0.73 |  |  |  |  |  |
| Hue | 102.81 | 101.53 | 95.07 | 101.32 | 99.03 | 100.91 | 101.16 | 101.49 | 7.59 | 0.48 |  |  |  |  |  |
| Chroma | 7.84 | 7.47 | 8.62 | 7.9 | 8.17 | 8.47 | 8.27 | 8.10 | 2.03 | 0.75 |  |  |  |  |  |

RMSE = root mean square error

^a,b,c^ different superscripts within row indicate significant differences between myopathy classes (*P* ≤ 0.05; Tukey test).

Supplementary Table 2. Breast physical parameters (least square mean) according to myopathy class. Control (CO), white striping (WS), wooden breast (WB), spaghetti meat (SM), and combinations WB-WS, WB-SM, SM-WS and WB-SM-WS were classified at global breast level.

|  | | CO | | WS | WB | SM | WB-WS | WB-SM | SM-WS | WB-SM-WS | RMSE | *P* value |
| --- | --- | --- | --- | --- | --- | --- | --- | --- | --- | --- | --- | --- |
| *Breasts (n)* | | *41* | | *31* | *7* | *52* | *22* | *15* | *42* | *30* |  |  |
|  |  | | Cranial region color | | | | | | | | | |
| L* | | 55.71^a^ | | 56.01^ab^ | 58.08^ab^ | 56.65^ab^ | 57.74^b^ | 56.10^ab^ | 56.99^ab^ | 57.42^ab^ | 2.31 | <0.01 |
| a* | | -0.54 | | -0.14 | 0.13 | -0.37 | 0.15 | -0.25 | -0.54 | -0.13 | 0.98 | 0.15 |
| b* | | 9.49^a^ | | 10.52^ab^ | 13.24^cd^ | 10.87^b^ | 13.28^d^ | 11.92^bcd^ | 11.32^bc^ | 11.79^bc^ | 1.51 | <0.001 |
| Hue | | 93.81^b^ | | 91.41^ab^ | 87.93^ab^ | 92.08^ab^ | 88.40^a^ | 91.86^ab^ | 92.97^ab^ | 91.07^ab^ | 5.43 | <0.05 |
| Chroma | | 9.54^a^ | | 10.54^ab^ | 12.32^bcd^ | 10.84^bc^ | 12.36^d^ | 11.94^bcd^ | 11.36^bcd^ | 11.91^cd^ | 1.35 | <0.001 |
|  |  | | Central region color | | | | | | | | | |
| L* | | 54.96 | | 54.97 | 56.87 | 55.41 | 56.51 | 56.18 | 55.44 | 55.49 | 2.28 | 0.13 |
| a* | | -1.49^a^ | | -1.19^a^ | -0.87^a^ | -1.45^a^ | -1.07^a^ | -1.34^a^ | -1.43^a^ | -0.98^a^ | 0.70 | <0.05 |
| b* | | 6.99^a^ | | 7.72^ab^ | 8.19^b^ | 7.55^ab^ | 8.37^b^ | 8.29^ab^ | 7.74^ab^ | 7.84^ab^ | 1.46 | <0.05 |
| Hue | | 102.27^a^ | | 98.85^a^ | 96.55^a^ | 101.69^a^ | 98.68^a^ | 100.51^a^ | 101.32^a^ | 98.00^a^ | 5.99 | <0.05 |
| Chroma | | 7.21^a^ | | 7.91^ab^ | 8.36^ab^ | 7.77^ab^ | 8.50^b^ | 8.50^ab^ | 8.01^ab^ | 8.05^ab^ | 1.46 | <0.05 |
|  |  | | Caudal region color | | | | | | | | | |
| L* | | 55.67 | | 54.71 | 56.98 | 56.06 | 56.06 | 54.88 | 55.35 | 54.85 | 2.46 | 0.13 |
| a* | | -1.34 | | -1.52 | -1.69 | -1.46 | -1.66 | -1.38 | -1.46 | -1.44 | 0.62 | 0.66 |
| b* | | 7.38 | | 7.05 | 7.95 | 7.74 | 8.04 | 7.89 | 7.94 | 7.42 | 1.30 | 0.06 |
| Hue | | 101.12 | | 103.05 | 102.54 | 101.41 | 102.78 | 100.80 | 101.14 | 102.20 | 5.63 | 0.80 |
| Chroma | | 7.54 | | 7.29 | 8.21 | 7.97 ^a^ | 8.28 | 8.10 | 8.19 | 7.72 | 1.31 | 0.06 |
| pH | | 5.78^a^ | | 5.87^ab^ | 6.09^c^ | 5.78^a^ | 6.00^c^ | 5.97^bc^ | 5.87^ab^ | 6.05^c^ | 0.15 | <0.001 |
| EC | | 8.12^ab^ | | 8.05^ab^ | 8.58^ab^ | 7.73^a^ | 9.61^b^ | 9.16^ab^ | 7.49^a^ | 8.72^ab^ | 1.95 | <0.001 |
| Drip loss (%) | | 1.65 | | 1.41 | 2.44 | 1.84 | 2.25 | 1.99 | 1.60 | 1.56 | 1.06 | 0.06 |
| Thawing loss (%) | | 11.54^bc^ | | 10.29^abc^ | 11.14^abc^ | 11.82^c^ | 9.28^ab^ | 9.30^abc^ | 11.50^bc^ | 8.45^a^ | 2.84 | <0.001 |
| Cooking loss (%) | | 25.40^a^ | | 26.10^a^ | 31.44^bc^ | 26.72^a^ | 31.85^c^ | 30.06^bc^ | 26.91^a^ | 29.09^b^ | 2.70 | <0.001 |
|  |  | | Warner-Brazler texture | | | | | | | | | |
| Maximum force (N) | | 17.80^b^ | | 16.32^abc^ | 15.05^ab^ | 17.13^bc^ | 14.54^ab^ | 14.34^ab^ | 16.57^bc^ | 13.88^a^ | 4.21 | <0.001 |
| Total force (N×mm) | | 62.82^b^ | | 56.563^b^ | 46.80^ab^ | 60.36^ab^ | 52.02^ab^ | 49.70^ab^ | 57.21^ab^ | 47.74^a^ | 17.11 | <0.001 |
| Slope force (N/mm) | | 3.07 | | 2.88 | 3.08 | 3.02 | 2.73 | 2.73 | 2.93 | 2.61 | 0.71 | 0.16 |

RMSE = root mean square error; EC = electrical conductivity

^a,b,c^ different superscripts within row indicate significant differences between myopathy classes (*P* ≤ 0.05; Tukey test).

Supplementary Table 3. Breast chemical composition (least square mean) according to myopathy class. Control (CO), white striping (WS), wooden breast (WB), spaghetti meat (SM), and combinations WB-WS, WB-SM, SM-WS and WB-SM-WS were classified at global breast level.

|  | CO | WS | WB | SM | WB-WS | WB-SM | SM-WS | WB-SM-WS | RMSE | *P* value |
| --- | --- | --- | --- | --- | --- | --- | --- | --- | --- | --- |
| *Breasts (n)* | *41* | *31* | *7* | *52* | *22* | *15* | *42* | *30* |  |  |
| Moisture (g/100g) | 76.34^a^ | 76.50^a^ | 79.28^c^ | 76.65^a^ | 78.42^bc^ | 78.02^bc^ | 76.89^a^ | 7.63^b^ | 0.90 | <0.001 |
| Protein (g/100g) | 21.64^b^ | 21.07^b^ | 18.52^a^ | 21.33^b^ | 18.97^a^ | 19.60^a^ | 21.00^b^ | 19.71^a^ | 0.96 | <0.001 |
| Fat (g/100g) | 1.26^ab^ | 1.60^cd^ | 1.44^abcd^ | 1.15^a^ | 1.77^cd^ | 1.44^abc^ | 1.45^bc^ | 1.91^d^ | 0.38 | <0.001 |
| Collagen (mg/g) | 4.42^ab^ | 4.49^ab^ | 5.75^c^ | 4.11^a^ | 5.40^c^ | 5.00^bc^ | 4.49^ab^ | 5.06^bc^ | 0.93 | <0.001 |

RMSE = root mean square error

^a,b,c^ different superscripts within row indicate significant differences between myopathy classes (*P* ≤ 0.05; Tukey test).
